# Supplementary material for: Burnout in Intensive Care Unit Workers during the Second Wave of the COVID-19 Pandemic: A Single Center Cross-Sectional Italian Study
Source: Int J Environ Res Public Health. 2021 Jun 5;18(11):6102. doi: 10.3390/ijerph18116102 (PMC8201217; doi:10.3390/ijerph18116102)
Supplement: Supplementary file 1 [file ijerph-18-06102-s001.zip › ijerph-1231333-supplementary.pdf]

Supplementary material, Table S1. Emotional exhaustion sub score (EE) by severity categories (Reference=Low/Medium)

	Low and Medium (N=80)	%	High (N=56)	%	OR or F	CI 95% or p
**Age:**						
< 35	27	*33.8*	27	*48.2*	*1.83*	*0.91-3.68*
≥35	53	*66.3*	29	*51.8*	*Ref*	
Missing	-					
**Age***	80; 40.1; 38 (23-68)		56; 37.8; 35 (24-58)		*1.73*	*0.1904*
Missing	-		-			
**Sex:**						
Male	38	*48.7*	17	*30.4*	*0.46*	*0.22-0.94*
Female	40	*51.3*	39	*69.6*	*Ref*	
Missing	*2*		-			
**Profession:**						
Nurse	49	*61.3*	35	*62.5*	*Ref*	
Physician	31	*38.8*	21	*37.5*	*0.95*	*0.47-1.92*
Missing	-		-			
**Living alone:**						
Yes	13	*19.4*	17	*34.7*	*2.21*	*0.95-5.13*
No	54	*80.6*	32	*65.3*	*Ref*	
Missing	*13*		*7*			
**Worry about transmitting the virus to the people you live with:**						
No/a little	23	*44.2*	10	*33.3*	*0.63*	*0.25-1.61*
A lot	29	*55.8*	20	*66.7*	*Ref*	
Missing	*2*		*2*			
**Years of work experience**	69; 13.8;10 (0-42)		45;13.6;12 (0-36)		*0.01*	*0.9386*
Missing	11		11			
**Work area before the pandemic:**						
ICU	48	*63.2*	39	*73.6*	*1.62*	*0.75-3.50*
Operating room/ surgery/ Other	28	*36.8*	14	*26.4*	*Ref*	
Missing	*4*		*3*			
**Difficulties in adapting to the new work environment:**						
Not at all	41	*53.9*	16	*33.3*	*Ref*	
Yes	35	*46.1*	32	*66.7*	*2.34*	*1.11-4.96*
Missing	*4*		*8*			
**Compared to before the Covid-19 emergency, the patient number/ workload has:**						

Increased	38	53.5	35	64.8	Ref	
Equal/diminished	33	46.5	19	35.2	0.62	0.30-1.29
Missing	9		2			
**Compared to before the Covid-19 emergency, how do you evaluate the relationship with your colleagues?**						
Improved	45	61.6	26	51.0	Ref	
Equal/ Got worse	28	38.4	25	49.0	1.54	0.75-3.19
Missing	7		5			
**Compared to before the Covid emergency, there are opportunities to discuss important decisions in groups:**						
More frequent	36	48.0	24	48.0	Ref	
Equal/ Less frequent	39	52.0	26	52.0	1.00	0.49-2.05
Missing	5		6			
**Do you feel protected when you work?**						
No /a little bit	25	31.6	30	57.7	2.94	1.42-6.09
Protected	54	68.4	22	42.3	Ref	
Missing	1		4			
**Hospital anxiety and depression scale:**						
**Anxiety**						
Normal anxiety	51	69.9	9	16.4	Ref	
Presence of symptoms of anxiety	22	30.1	46	83.6	11.84	4.95-28.33
Missing	7		1			
**Depression**						
Normal depression	59	77.6	10	20.0	Ref	
Presence of symptoms of depression	17	22.4	40	80.0	13.88	5.77-33.41
Missing	4		6			
**Resilience**						
Low or very low	8	10.0	16	28.6	3.60	1.42-9.15
Moderate or high	72	90.0	40	71.4	Ref	
Missing						
**Insomnia**						
Clinical insomnia	33	47.1	42	80.8	4.71	2.04-10.84
No clinically significant insomnia	37	52.9	10	19.2	Ref	
Missing	10		4			

** N.; Mean; Median (Range)*

Supplementary material, Table S2. Depersonalization sub score (DP) by severity categories (Reference= Low/Medium)

	Low and Medium (N=71)	*%*	*High (N=65)*	*%*	*OR or F*	*CI 95% or p*
**Age:**						
< 35	24	33.8	30	46.2	1.68	0.84-3.35

≥35	47	*66.2*	35	*53.8*	*Ref*	
Missing	-		-			
**Age***	71; 39.9; 38 (23-58)		65; 38.3; 37 (23-68)		*1.00*	*0.3192*
Missing	-		-			
**Sex:**						
Male	28	*40.6*	27	*41.5*	*1.04*	*0.52-2.07*
Female	41	*59.4*	38	*58.5*	*Ref*	
Missing	*2*		-			
**Profession:**						
Nurse	38	*53.5*	46	*70.8*	*Ref*	
Physician	33	*46.5*	19	*29.2*	*0.48*	*0.23-0.97*
Missing	-		-			
**Living alone:**						
Yes	15	*25.0*	15	*26.8*	*1.10*	*0.48-2.52*
No	45	*75.0*	41	*73.2*	*Ref*	
Missing	*11*		*9*			
**Worry about transmitting the virus to the people you live with:**						
No/a little	23	*54.8*	25	*62.5*	*0.80*	*0.33-1.94*
A lot	19	*45.2*	15	*37.5*	*Ref*	
Missing	*3*		*1*			
**Years of work experience**	62;12.5;7 (0-34)		52;15.2;13.5 (0-41)		1.66	0.2006
Missing	*9*		13			
**Work area before the pandemic:**						
ICU	42	*61.8*	45	*73.8*	*1.74*	*0.82-3.69*
Operating room/ surgery/ Other	26	*38.2*	16	*26.2*	*Ref*	
Missing	*3*		*4*			
**Difficulties in adapting to the new work environment:**						
Not at all	32	*50.0*	25	*41.7*	*Ref*	
Yes	32	*50.0*	35	*58.3*	*1.40*	*0.69-2.85*
Missing	*7*		*5*			
**Compared to before the Covid-19 emergency, the patient number/ workload has:**						
Increased	45	*68.2*	28	*47.5*	*Ref*	
Equal/diminished	21	*31.8*	31	*52.5*	*2.37*	*1.15-4.91*
Missing	*5*		*6*			
**Compared to before the Covid-19 emergency, How do you evaluate the**						

**relationship with your colleagues?**						
Improved	39	*58.2*	32	*56.1*	*Ref*	
Equal/ Got worse	28	*41.8*	25	*43.9*	*1.09*	*0.53-2.22*
Missing	*4*		*8*			
**Compared to before the Covid-19 emergency, there are opportunities to discuss important decisions in groups:**						
More frequent	31	*44.9*	29	*51.8*	*Ref*	
Equal/ Less frequent	38	*55.1*	27	*48.2*	*0.76*	*0.37-1.54*
Missing	*2*		*9*			
**Do you feel protected when you work?**						
No /a little bit	24	*34.3*	31	*50.8*	*1.98*	*0.98-4.00*
Protected	46	*65.7*	30	*49.2*	*Ref*	
Missing	*1*		*4*			
**Hospital anxiety and depression scale:**						
**Anxiety**						
Normal anxiety	40	*58.8*	20	*33.3*	*Ref*	
Presence of symptoms of anxiety	28	*41.2*	40	*66.7*	*2.86*	*1.39-5.88*
Missing	*3*		*5*			
**Depression**						
Normal depression	46	*69.7*	23	*38.3*	*Ref*	
Presence of symptoms of depression	20	*30.3*	37	*61.7*	*3.70*	*1.77-7.75*
Missing	*5*		*5*			
**Resilience**						
Low or very low	8	*11.3*	16	*24.6*	*2.57*	*1.02-6.50*
Moderate or high	63	*88.7*	49	*75.4*	*Ref*	
Missing	-		-			
**Insomnia**						
Clinical insomnia	35	*55.6*	49	*83.1*	*1.68*	*0.80-3.52*
No clinically significant insomnia	28	*44.4*	19	*32.2*	*Ref*	
Missing	*8*		*6*			

** N.; Mean; Median (Range)*

Supplementary material, Table S3. Personal accomplishment sub score (PA) by severity categories (Reference=High/Medium)

.	**High/Medium (N=64)**	*%*	**Low (N=72)**	*%*	**OR or F**	**CI 95% or p**
**Age:**						
< 35	20	*31.3*	34	*47.2*	*1.97*	*0.97-3.97*
≥35	44	*68.8*	38	*52.8*	*Ref*	
Missing	-		-			

**Age***	64; 39.9; 38 (23-68)		72; 38.4; 36 (24-58)		*0.83*	*0.3634*
Missing	-		-			
**Sex:**						
Male	25	*40.3*	30	*41.7*	*1.06*	*0.53-2.11*
Female	37	*59.7*	42	*58.3*	*Ref*	
Missing	*2*		-			
**Profession:**						
Nurse	40	*62.5*	44	*61.1*	*Ref*	
Physician	24	*37.5*	28	*38.9*	*1.06*	*0.53-2.12*
Missing	-		-			
**Living alone:**						
Yes	11	*20.0*	19	*31.1*	*1.81*	*0.77-4.25*
No	44	*80.0*	42	*68.9*	*Ref*	
Missing	*9*		*11*			
**Worry about transmitting the virus to the people you live with:**						
No/a little	19	*44.2*	14	*35.9*	*0.71*	*0.29-1.72*
A lot	24	*55.8*	25	*64.1*	*Ref*	
Missing	*1*		*3*			
**Years of work experience**	59;13.8;12 (0-41)		55;13.7;9 (0-36)		0.01	0.9412
Missing	*5*		*17*			
**Work area before the pandemic:**						
ICU	44	*72.1*	43	*63.2*	*0.66*	*0.31-1.40*
Operating room/ surgery/ Other	17	*27.9*	25	*36.8*	*Ref*	
Missing	*3*		*4*			
**Difficulties in adapting to the new work environment:**						
Not at all	26	*45.6*	31	*46.3*	*Ref*	
Yes	31	*54.4*	36	*53.7*	*0.97*	*0.48-1.98*
Missing	*7*		*5*			
**Compared to before the Covid-19 emergency, the patient number/ workload has:**						
Increased	37	*61.7*	36	*55.4*	*Ref*	
Equal/diminished	23	*38.3*	29	*44.6*	*1.30*	*0.63-2.65*
Missing	*4*		*7*			
**Compared to before the Covid-19 emergency, how do you evaluate the relationship with your colleagues?**						

Improved	33	*58.9*	38	*55.9*	*Ref*	
Equal/ Got worse	23	*41.1*	30	*44.1*	*1.13*	*0.55-2.32*
Missing	*8*		*4*			
**Compared to before the Covid-19 emergency, there are opportunities to discuss important decisions in groups:**						
More frequent	28	*46.7*	32	*49.2*	*Ref*	
Equal/ Less frequent	32	*53.3*	33	*50.8*	*0.90*	*0.45-1.82*
Missing	*4*		*7*			
**Do you feel protected when you work?**						
No /a little bit	18	*28.6*	37	*54.4*	*2.98*	*1.44-6.16*
Protected	45	*71.4*	31	*45.6*	*Ref*	
Missing	*1*		*4*			
**Hospital anxiety and depression scale:**						
**Anxiety**						
Normal anxiety	35	*58.3*	25	*36.8*	*Ref*	
Presence of symptoms of anxiety	25	*41.7*	43	*63.2*	*2.41*	*1.18-4.91*
Missing	*4*		*4*			
**Depression**						
Normal depression	40	*67.8*	29	*43.3*	*Ref*	
Presence of symptoms of depression	19	*32.2*	38	*56.7*	*2.76*	*1.33-5.72*
Missing	*5*		*5*			
**Resilience**						
Low or very low	2	*3.1*	22	*30.6*	*13.64*	*3.06-60.80*
Moderate or high	62	*96.9*	50	*69.4*	*Ref*	
Missing	-		-			
**Insomnia**						
Clinical insomnia	37	*63.8*	38	*59.4*	*0.83*	*0.40-1.72*
No clinically significant insomnia	21	*36.2*	26	*40.6*	*Ref*	
Missing	*6*		*8*			

** N.; Mean; Median (Range)*
